# Calycosin prevents bone loss induced by hindlimb unloading

**DOI:** 10.1038/s41526-022-00210-x

**Published:** 2022-07-06

**Authors:** Xiang Jin, Hong Wang, Xuechao Liang, Kang Ru, Xiaoni Deng, Shuo Gao, Wuxia Qiu, Ying Huai, Jiaqi Zhang, Linbin Lai, Fan Li, Zhiping Miao, Wenjuan Zhang, Airong Qian

**Affiliations:** 1 Lab for Bone Metabolism, Xi’an Key Laboratory of Special Medicine and Health Engineering; Key Lab for Space Biosciences and Biotechnology, School of Life Sciences, Northwestern Polytechnical University, Xi’an, Shaanxi China; 2 Research Center for Special Medicine and Health Systems Engineering, School of Life Sciences, Northwestern Polytechnical University, Xi’an, Shaanxi China; 3 NPU-UAB Joint Laboratory for Bone Metabolism, School of Life Sciences, Northwestern Polytechnical University, Xi’an, Shaanxi China; 4 Research Center for toxicological and biological effects, Institute for Hygiene of Ordnance Industry, Xi’an, 710065 China; 5 Hospital of Northwestern Polytechnical University, Xi’an, Shaanxi 710072 China

**Keywords:** Screening, Pharmacology

## Abstract

Bone loss induced by microgravity exposure seriously endangers the astronauts’ health, but its countermeasures still have certain limitations. The study aims to find potential protective drugs for the prevention of the microgravity-induced bone loss. Here, we utilized the network pharmacology approach to discover a natural compound calycosin by constructing the compound-target interaction network and analyzing the topological characteristics of the network. Furthermore, the hind limb unloading (HLU) rats’ model was conducted to investigate the potential effects of calycosin in the prevention of bone loss induced by microgravity. The results indicated that calycosin treatment group significantly increased the bone mineral density (BMD), ameliorated the microstructure of femoral trabecular bone, the thickness of cortical bone and the biomechanical properties of the bone in rats, compared that in the HLU group. The analysis of bone turnover markers in serum showed that both the bone formation markers and bone resorption markers decreased after calycosin treatment. Moreover, we found that bone remodeling-related cytokines in serum including IFN-γ, IL-6, IL-8, IL-12, IL-4, IL-10 and TNF-α were partly recovered after calycosin treatment compared with HLU group. In conclusion, calycosin partly recovered hind limb unloading-induced bone loss through the regulation of bone remodeling. These results provided the evidence that calycosin might play an important role in maintaining bone mass in HLU rats, indicating its promising application in the treatment of bone loss induced by microgravity.

## Introduction

Bone loss induced by spaceflight has become one of the most important risk factors for astronauts, which threatens astronauts’ health and limits space exploration^1,2^. Among the 60 American and Russian astronauts who participated in the Mir Space Station (Mir) and the International Space Station (ISS) long-duration space missions (average 176 ± 45 days), about 92% of the astronauts suffered from more than 5% bone loss in at least one skeletal part, 40% of the astronauts suffered from more than 10% bone loss in at least one skeletal part^3^. When the Soviet astronauts flew in space for 75 to 184 days, the bone mineral density (BMD) of the calcaneus decreased 0.9–19.8%, and the spine BMD decreased about 0.3–10.8% for the astronauts who stayed at the Salute Space Station for 5 to 7 months^4^. At present, several preventative and therapeutic strategies have shown good efficacy for bone loss induced by spaceflight, such as physical exercise, mechanical stimulation and drug therapy^5,6^, but still have certain limitations and significant deficiencies. Among them, drug treatments such as bisphosphonates have significant protective effects on bone loss induced by microgravity, but there were the inevitable side effects, such as osteonecrosis of the jaw, headache and nausea^7–9^. Therefore, new safer compounds could be found to prevent and treat bone loss induced by spaceflight.

Recent studies demonstrated that natural small molecule drugs derived from Traditional Chinese Medicine (TCM) for homology of medicine and food, have good prospects in the treatment of osteoporosis because of their safety, low side effects and low cost, such as curcumin, lycopene, and resveratrol, etc^10–12^. Radix Astragali, as a kind of TCM for homology of medicine and food, has been reported to have a positive therapeutic effect on bone disorder after spinal cord injury^13^. However, due to the complexity of its ingredients and the limitations of traditional drug research methods, it remains difficult to find the effective compounds for the treatment of bone loss. At present, the network pharmacology has been applied to comprehensively determine the potential active compounds and targets of complex drugs^14,15^. Network pharmacology integrates network biology analysis, gene connectivity and redundancy, and gene pleiotropy on the basis of systems biology and multi-directional pharmacology^16^, which provides a new idea for drug discovery and mechanism research^17,18^.

In this study, we initially used the network pharmacology to identify the effective natural compounds of *Radix Astragali* for the prevention of bone loss. Furthermore, the HLU rat model was used to evaluate the function of the compounds screened by network pharmacology analysis in bone loss. As a result, we found that calycosin partly recovered HLU-induced bone loss through the regulation of bone remodeling. This research will provide a promising candidate for the treatment of bone loss induced by spaceflight.

## Methods

### Active compounds and targets of Radix Astragali

The chemical components of the herb in *Radix Astragali* were determined through the Traditional Chinese Medicine Systems Pharmacology Database and Analysis Platform (TCMSP, https://tcmspw.com/tcmsp.php): a common database for the study of TCM and the components of Chinese herbal medicine. To further obtain the effective compounds from *Radix Astragali*, we performed an in silico ADME approach, which integrated predict drug-likeness (PreDL), predict oral bioavailability (PreOB) and predict Caco-2 permeability (PreCaco-2). Oral bioavailability (OB), as one of the most important pharmacokinetic parameters, can determine whether a small molecule compound has drug activity. Herein, we employed an in-house system OBioavail1.1 to perform the OB screening^19^. Additionally, Caco-2 permeability prediction model PreCaco-2 was developed to predict absorption of the drug through a robust in silico. And the PreDL was developed to classify drug-like and nondrug-like chemicals by the molecular descriptors and Tanimoto coefficient (as shown in Eq. (1))^20^.

1 $${{{\mathrm{T(A,B) = }}}}\frac{{{{{\mathrm{A}}}} \cdot {{{\mathrm{B}}}}}}{{\left| {{{\mathrm{A}}}} \right|^2 + \left| {{{\mathrm{B}}}} \right|^2 - {{{\mathrm{A}}}} \cdot {{{\mathrm{B}}}}}}$$

where A denotes the molecular properties of ingredients in herb, and B represents the average drug-likeness index of all compounds in DrugBank database (http://www.drugbank.ca/). In this work, the compounds matching OB ≥ 30%, DL ≥ 0.18 and Caco-2 ≥ -0.4 were screened as bioactive compounds.

Further, we conduct the prediction and discovery of the targets of these active compounds based on a new computational model termed SysDT through two powerful algorithms of Random Forest (RF) and Support Vector Machine (SVM), which integrates large-scale information of chemistry, genomics and pharmacology^21^. Subsequently, we individually convert these predicted target names into the standard gene symbols through the Uniprot database (https://www.uniprot.org).

### Disease target

The targets associated to bone loss, were identified from the GeneCards database (https://www.genecards.org/), using “osteoporosis” as the search term, and all the genes found in the database were considered as the target set of osteoporosis.

### Network construction and topological analysis

To analyze and find potential drugs in the treatment of bone loss, we constructed and analyzed the visualized compound-target (C-T) network in this study. Using all bioactive compounds of *Radix Astragali* and the common targets of these active compounds with bone loss disease, a network diagram of compound-target interactions was generated, in which a compound and a target were linked with each other. By analyzing the topological properties of the C-T network, we identified the key compounds according to the degree values and neighborhood connectivity of nodes. The visualization of the network was achieved by the Cytoscape software.

### Animal experiments

Twenty-four healthy Sprague-Dawley male rats weighing 200 ± 20 g (SPF standard) were kept at a suitable temperature of 25 ± 3 °C with a 12 h light-dark cycle. After 7 days of adaptation, all rats were randomly divided into four groups with six rats in each group, including baseline group, ground control group, HLU group, HLU treatment group with calycosin, 30 mg· kg^−1^ ·day^−1^. Among them, the rats in the baseline group were sacrificed before hind limb unloading and preserved their tissues for further examination. The rats in the control group were free to move without HLU, but the rats tails in the HLU and HLU+Calycosin groups were suspended to unload their hindlimbs as suggested by MoreyHolton and Globus^22^. Calycosin was intragastrically administered once a day in the HLU+Calycosin group for 4 weeks; and an equal volume of 0.5% CMC-Na was received in the control and HLU groups, daily, for 4 weeks. Besides, the rats of each group were injected with 5 mg/kg calcein subcutaneously on days 13 and 3 before necropsy. All procedures strictly followed the Guidelines for the Care and Use of Laboratory Animals, and were approved by the Institutional Ethics Committee of Northwestern Polytechnical University.

### BMD analysis

After the rats were injected intraperitoneally with anesthesia and deeply anesthetized, they were neatly arranged on test bench of the bone densitometer. We scanned the whole body of the rats by the small animal scan mode using dual-energy X-ray absorptiometry (DEXA) assay (Lunar Prodigy Advance DXA, GE healthcare, Madison, WI, USA)^23^. After all scans were completed, then we selected the femurs as the region of interest (ROI) to analyze the BMD value using GE’s purpose-designed software (enCORE 2006, GE Healthcare, Madison, WI, USA).

### Micro-CT scanning

The right femur fixed with 4% paraformaldehyde (PFA) was fixed on the template and scanned along the long axis with a micro CT scanner (Skyscan 1276, Bruker microCT, Kontich, Belgium)^24^. The specific parameters during the scanning: voltage was 70 kVp, current was 114 μA, the image pixel was 10 μm, and layer spacing was 10 μm. Later, the scanning files were analyzed to reconstruct and analyze the three-dimensional image. And the trabecular bone with a 2 mm thickness and 1 mm in the growth plate was selected as the area of interest for further data analysis. The 3D parameters for qualitative analysis of the trabecular bone were as follows: the bone volume per tissue volume (BV/TV; %), trabecular number (Tb.N; 1/mm), trabecular spacing (Tb.Sp; mm), trabecular thickness (Tb.Th; mm), and trabecular pattern factor (Tb.Pf; 1/mm). The cortical bone with a 0.5 mm thickness and 4 mm below the femoral growth plate was selected as the region of interest for further data analysis. The 3D parameters for qualitative analysis of the cortical bone were the cortical thickness (Cr.Th; mm). Among them, the 3D parameters of the trabecular bone and cortical bone were qualitatively analyzed by CTan software, and the 3D images of the trabecular bone and cortical bone were constructed by CTvol software and CTan software (Skyscan 1276, Bruker microCT, Kontich, Belgium).

### Three-point bending mechanical test

The mechanical properties of the left femurs were tested using a universal material testing machine (Intron company, USA). Before mechanical testing, the left femurs stored at −80 °C were soaked in saline solution to thaw for about 4 h, and then measured them on the material testing machine. Each sample was placed on two supports separated by a distance of 20 mm. The load was applied to the middle of the diaphysis and pressed down at a speed of 2 mm/min until fracture occurred^25^. The load and displacement data were then recorded by acquisition computer. According to the load-displacement curve and the internal and external diameters of the fractured bone, the mechanical properties were calculated by Matlab software, including: the maximum load (N), toughness (J/mm^2^), stiffness (N/mm), maximum stress (MPa) and Young’s modulus (GPa).

### Dynamic bone histomorphometry

The femurs were fixed in 4% PFA and dehydrated in 60–70–80–90–95% alcohol, with each step taking for 24 to 72 h. It was then dehydrated twice with for 12 h to 24 h each time, as was xylene. Next, the femurs were put into the embedding bottle, and adding an appropriate amount of embedding agent. Routine sectioning about 10 µm by the hard tissue slicer (Leica Microtome, HistoCore AUTOCUT) and place sections in an oven at 60 °C overnight. The dynamic parameters of mineral deposition rate (MAR) (micrometers per day) were quantified using CaseViewer software.

### Serum analysis

The rats’ blood was collected from the abdominal aorta. Serum was obtained by centrifuging at 1200 rpm for 10 min at 4 °C. The concentrations of bone turnover markers and cytokines of bone remodeling in serum were measured by using rat ELISA kits, including NTX, TRACP5b, PINP, RANKL, OPG, BGP, ALP, IL-4, IL-6, IL-8, IL-10, IL-12, TNF-α and IFN-γ. All testing procedures were carried out according to the manufacturer’s instructions.

### Statistical analysis

All the results are presented as the mean ± SD. Statistical analyses were carried out using GraphPad Prism 8.0 (GraphPad Software Inc., San Diego, CA, USA). Statistical significant differences were performed by two-tailed Student’s t test analysis between two groups. *P* < 0.05 was considered statistically significant.

### Reporting summary

Further information on research design is available in the Nature Research Reporting Summary linked to this article.

## Results

### The screening of active compounds in Radix Astragali

In order to screen the potential compounds in *Radix Astragali*, we initially collected all the compounds through the TCMSP database, and then a systematic pharmacology model, combining the absorption, distribution, metabolism, and excretion (ADME) screening model, were further employed^26^. In total, 87 compounds were collected from the *Radix Astragali* (as displayed in Supplementary Table 1). The ADME qualities of each component, including oral bioavailability (OB), drug-likeness (DL), and Caco-2 permeability (Caco-2), were to be examined in order to further obtain potential natural products with desirable pharmacological properties. As a result, 16 potential bioactive compounds with OB ≥ 30%, DL ≥ 0.18 and Caco-2 ≥ −0.40 were screened out, accounting for 18.4% of all 87 ingredients of *Radix Astragali*. Detailed information of the compounds is presented in Supplementary Table 2.

### Intersection of drug-disease targets

**Fig. 1:**
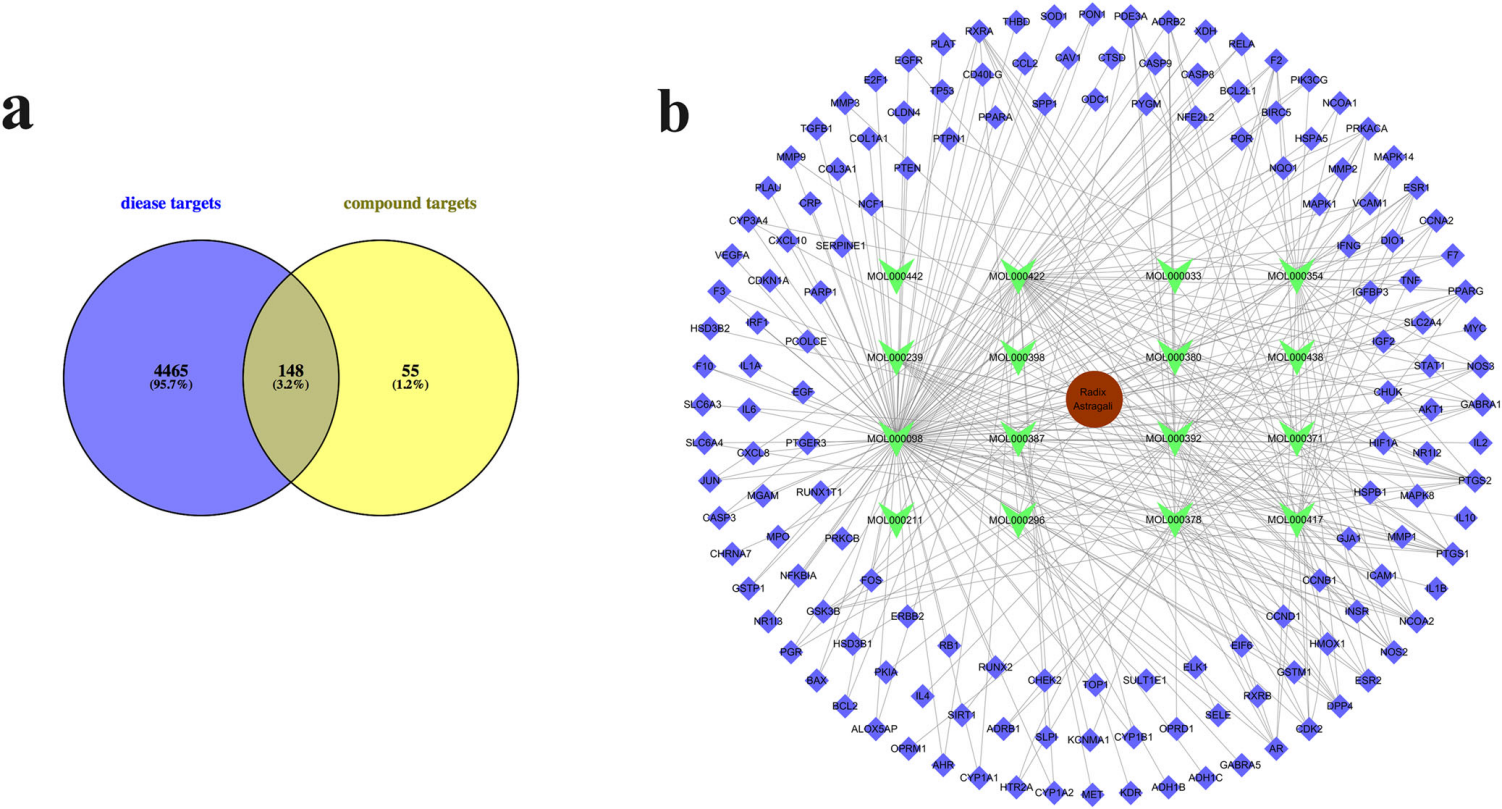
The analysis of Network pharmacology. **a** Venn diagram of targets for active compounds of *Radix Astragali* and osteoporosis-related targets. **b** The visualization network of compound-target was built by Cytoscape 3.8.0. It is composed of 148 target nodes (diamond, purple), 16 compound nodes (quadrilateral, green) and 1 Chinese herbal medicine node (round, red).

To obtain the potential targets of each compound in *Radix Astragali* for the treatment of bone loss, we took the intersection of compound targets and bone disease targets. Totally, we obtained 203 candidate targets of 16 compounds targets and 4613 targets for the disease based on the SysDT model and Genecards database, respectively. The construction of Venn diagram revealed a total of 148 targets as the common target genes between compounds and osteoporosis disease, as shown in Fig. 1a and Supplementary Table 3. In addition, a majority of natural products were shown to be hit by numerous targets, indicating that the multiple pharmacological effects of these compounds. For instance, calycosin (MOL000417) can interact with 23 targets, and Jaranol (MOL000239) targeted on 8 different proteins related to osteoporrosis. Among them, the information of 23 interacting targets of calycosin related to osteoporosis are shown in Table 1.

**Table 1:** The information of 23 interacting targets of calycosin related to osteoporosis.

	Target name	Name	Uniprot ID	Organism
1	ADRA1D	Alpha-1D adrenergic receptor	P25100	Homo sapiens
2	ADRA2C	Alpha-2C adrenergic receptor	P18825	Homo sapiens
3	ADRB1	Beta-1 adrenergic receptor	P08588	Homo sapiens
4	HTR3A	5-hydroxytryptamine receptor 3A	P46098	Homo sapiens
5	ACHE	Acetylcholinesterase	P22303	Homo sapiens
6	ADRA1B	Alpha-1B adrenergic receptor	P35368	Homo sapiens
7	ADRB2	Beta-2 adrenergic receptor	P07550	Homo sapiens
8	CAMKK2	Calmodulin	Q96RR4	Homo sapiens
9	PDE3A	CGMP-inhibited 3’,5’-cyclic phosphodiesterase A	Q14432	Homo sapiens
10	ESR1	Estrogen receptor	P03372	Homo sapiens
11	GABRA1	Gamma-aminobutyric acid receptor subunit alpha-1	P14867	Homo sapiens
12	CHRM1	Muscarinic acetylcholine receptor M1	P11229	Homo sapiens
13	CHRM3	Muscarinic acetylcholine receptor M3	P20309	Homo sapiens
14	NOS2	Nitric oxide synthase, inducible	P35228	Homo sapiens
15	NOS3	Nitric oxide synthase, endothelial	P29474	Homo sapiens
16	NCOA2	Nuclear receptor coactivator 2	Q15596	Homo sapiens
17	PTGS1	Prostaglandin G/H synthase 1	P23219	Homo sapiens
18	PTGS2	Prostaglandin G/H synthase 2	P35354	Homo sapiens
19	RXRA	Retinoic acid receptor RXR-alpha	P19793	Homo sapiens
20	SCN5A	Sodium channel protein type 5 subunit alpha	Q14524	Homo sapiens
21	F2	Prothrombin	P00734	Homo sapiens
22	PRSS1	Trypsin-1	P07477	Homo sapiens
23	OPRM1	Mu-type opioid receptor	P35372	Homo sapiens

### Building of compound-target network to screen key compound

To further determine the key compounds in the treatment of bone loss, a compound-target (C-T) interaction network was constructed and analyzed by Cytoscape software. As shown in Fig. 1b, the compound-target interaction network was built, which including 149 targets and 16 compounds. The C-T interaction network visually displayed the regulatory relationship between the compounds and targets. As shown in Supplementary Table 2, the topology analysis of the network displayed that quercetin (MOL000098, degree = 118), kaempferol (MOL000422, degree = 44), 7-O-methylisomucronulatol (MOL000378, degree = 29), formononetin (MOL000392, degree = 28), isorhamnetin (MOL000354, degree = 26) and calycosin (MOL000417, degree = 24) exhibited the higher degree number of interactions with various protein targets. Among these 6 compounds with higher degree values, calycosin showed the highest neighborhood connectivity value. Therefore, we speculated that calycosin might be the key compound in the treatment of bone loss.

### Effects of calycosin on bone microstructure and BMD

**Fig. 2:**
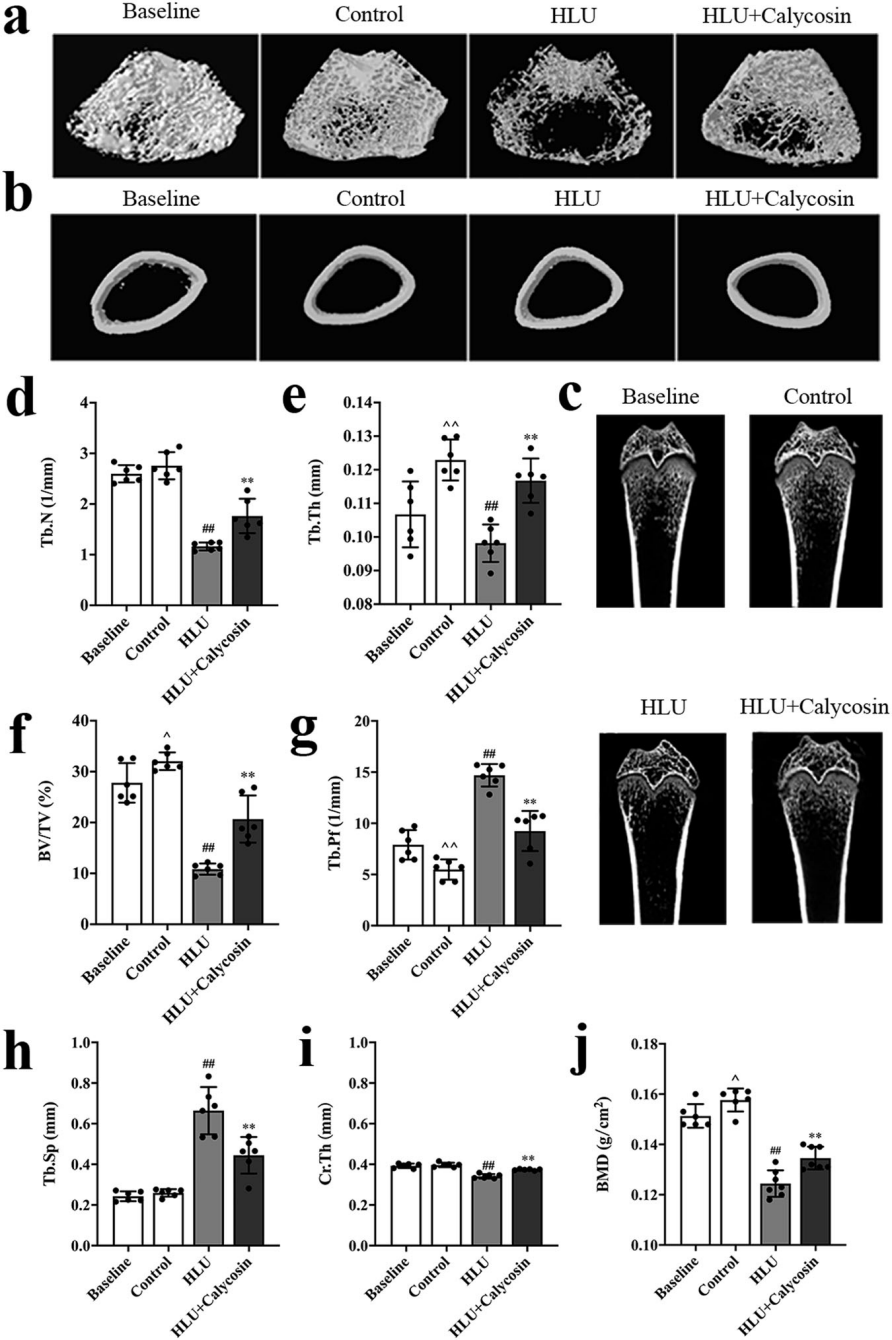
Effects of calycosin on bone microstructure and BMD. Representative Micro-CT 3D images of trabecular bone of distal femurs (**a**) and cortical bone of diaphyseal femurs (**b**) within various groups. **c** Representative section images of trabecular bones of distal femurs. **d** Quantitative analysis of the trabecular number (Tb.N) (**d**), trabecular thickness (Tb.Th) (**e**), bone volume per tissue volume (BV/TV) (**f**), trabecular bone pattern factor (Tb.Pf) (**g**), trabecular spacing (Tb.Sp) (**h**), cortical thickness (Cr.Th) (**i**) in cortical bone and BMD (**j**) in trabecular bone. Here and in succeeding bar charts with dot, the centre line of the whiskers, which is also the upper bound of the box, depicts the group mean, and the whiskers correspond to the standard deviation (SD). The results are represented as the mean ± SD; *n* = 6/group. *P*-values calculated using a two-tailed Student’s *t* test (^*P* < 0.05, ^^*P* < 0.01 *v*.*s*. baseline; ^#^*P* < 0.05, ^##^*P* < 0.01 v.s. control; *P < 0.05, **P < 0.01 v.s. HLU).

Based on the above results of network pharmacology, a natural bioactive compound calycosin for the protection against bone loss was screened out. To directly investigate the effects of calycosin against bone loss in vivo, the HLU rat model was employed to mimic the bone loss induced by spaceflight microgravity. The microarchitectural changes in the femoral diaphysis were assessed using Micro-CT, as a measure of bone quality. The representative three-dimensional (3D) images of the cancellous bone and cortical bone of the femoral metaphysis in the right femur are shown in Fig. 2a, b, respectively. The trabecular Tb. Th, BV/TV and femurs bone mineral density (BMD) of the control group were increased compared to baseline and the trabecular Tb. Pf in the control group were decreased compared with baseline group, indicating that the skeleton grows in the normal environment during 4 weeks (Fig. 2e–g, j). The trabecular bones of the control group are tightly connected and the mesh structure is complete. Compared with the control group, the trabecular bone parameters BV/TV, Tb. Th, Tb. N were significantly decreased and Tb. Sp, Tb. Pf were significantly increased in the HLU group (*P* < 0.01), indicating that hind limb unloading caused severe damage to the cancellous bone (Fig. 2d–h). However, the trabecular bone parameters of BV/TV, Tb. Th and Tb. N were significantly increased by calycosin treatment (*P* < 0.01) and the Tb. Sp, Tb. Pf were significantly reduced compared with HLU rats (*P* < 0.01), indicating that the microarchitectural properties were improved after treatment with calycosin. In addition, the analysis of cortical bone characteristics in the diaphyseal region showed that HLU caused a reduction of cortical bone thickness significantly (*P* < 0.01), whereas the treatment of HLU rats with calycosin significantly increased the cortical bone thickness (*P* < 0.01) (Fig. 2i). The changes of BMD after various treatments are shown in Fig. 2j. After hind limb unloading, the BMD of the HLU group were decreased significantly compared with that in the control group (*P* < 0.01), indicating HLU gave rise to the reduction of bone mass in rats. Rats treated with calycosin obviously increased the BMD values compared with HLU rats (*P* < 0.01), indicating that calycosin might play an important role in maintaining bone mass of tail suspended rats.

### Effect of calycosin on bone biomechanics

**Fig. 3:**
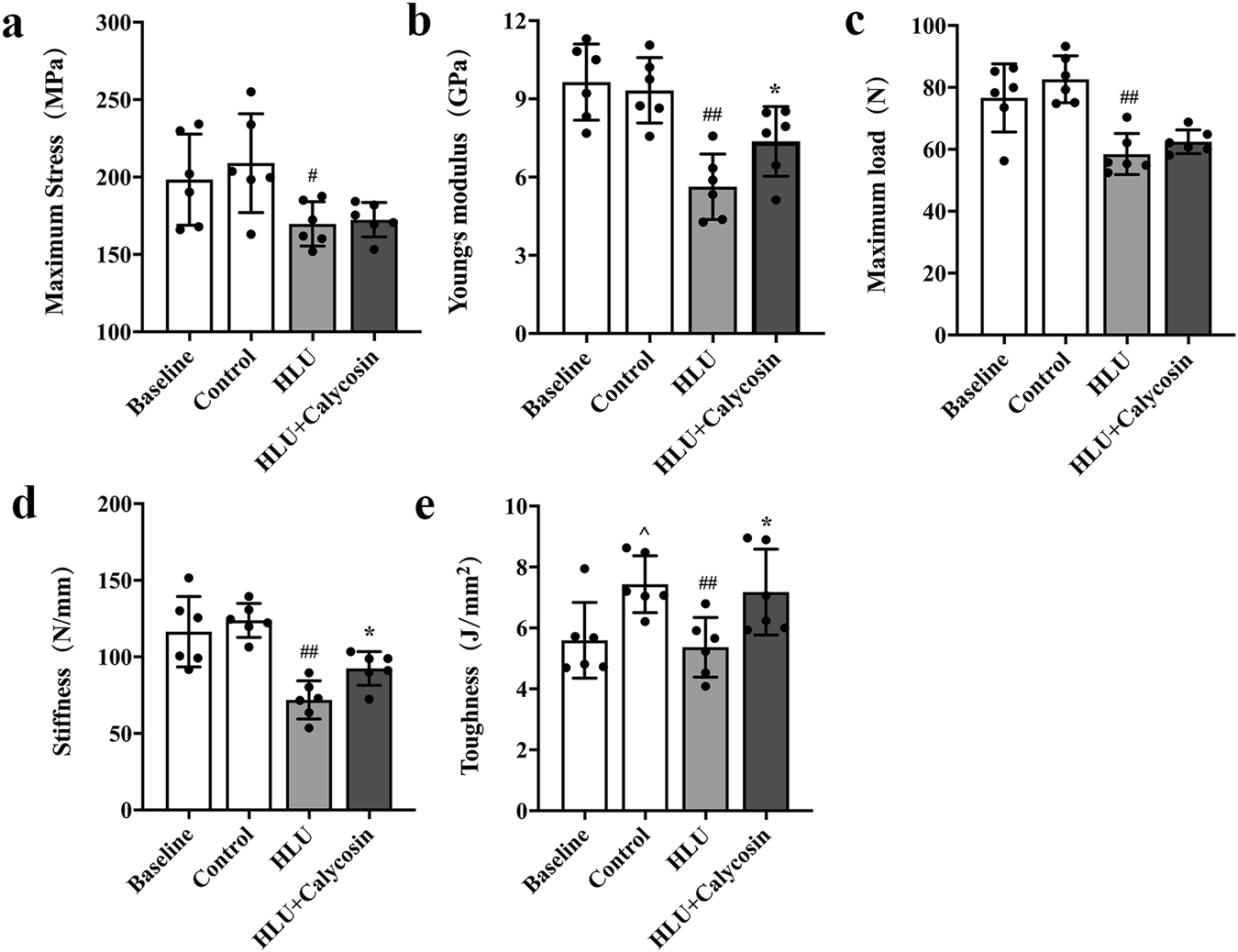
Effect of calycosin on bone biomechanics. Evaluation parameters in the four groups of rats included the following: maximum stress (**a**), Young’s modulus (**b**), maximum load (**c**), stiffness (**d**), and toughness (**e**). The results are represented as the mean ± SD; *n* = 6/group. *P*-values calculated using a two-tailed Student’s *t* test (^*P* < 0.05, ^^*P* < 0.01 v.s. baseline; ^#^*P* < 0.05, ^##^*P* < 0.01 v.s. control; *P < 0.05, **P < 0.01 v.s. HLU).

To further evaluate the effect of calycosin on the biomechanical properties, we performed a three-point bending test on the mid-diaphysis femurs of rats. The results of three-point bending test are shown in Fig. 3. Compared with the control group, the biomechanical parameters of maximum stress, maximum load, stiffness, Young’s modulus and toughness in the HLU group were obviously decreased (*P* < 0.05 or *P* < 0.01) (Fig. 3a–e). Furthermore, treatment with calycosin significantly prevented the HLU-induced decrease of Young’s modulus, toughness and stiffness (*P* < 0.05), while the maximum stress and maximum load did not change notably (*P* > 0.05).

### Effect of calycosin on bone histomorphometry

**Fig. 4:**
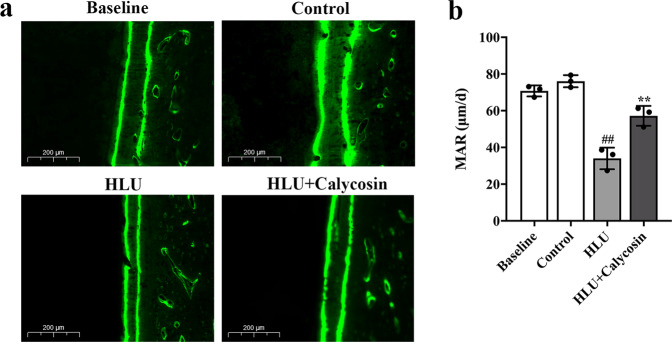
Effect of calycosin on bone histomorphology. **a** Representative fluorescence micrographs of trabecular bone sections showing green calcein labels within various groups (scale bar: 200 μm). **b** Quantitative analysis of the mineral deposition rate (MAR). The results are represented as the mean ± SD; *n* = 3/group. *P*-values calculated using a two-tailed Student’s t test (^*P* < 0.05, ^^*P* < 0.01 *v.s*. baseline; ^#^*P* < 0.05, ^##^*P* < 0.01 v.s. control; *P < 0.05, **P < 0.01 v.s. HLU).

Bone histomorphology can dynamically reflect the histophysiological characteristics and changes of bone, and accurately depict the pathological dynamics process of bone repair and reconstruction. The distance between two labeled calcein fluorescence lines in bone tissue was monitored dynamically in this work to determine bone formation. Figure 4a shows the representative images of bone deposition line in each group. As shown in Fig. 4b, further quantitative analysis of calcein double labeling demonstrated that the rats in the HLU group displayed an obviously lower mineral apposition rate (MAR) compared with that in the control group (*P* < 0.01). Yet, calycosin administration increased the rate of calcification in the HLU + Calycosin group compared with that in the HLU group (*P* < 0.01).

### Effect of calycosin on bone turnover markers

**Fig. 5:**
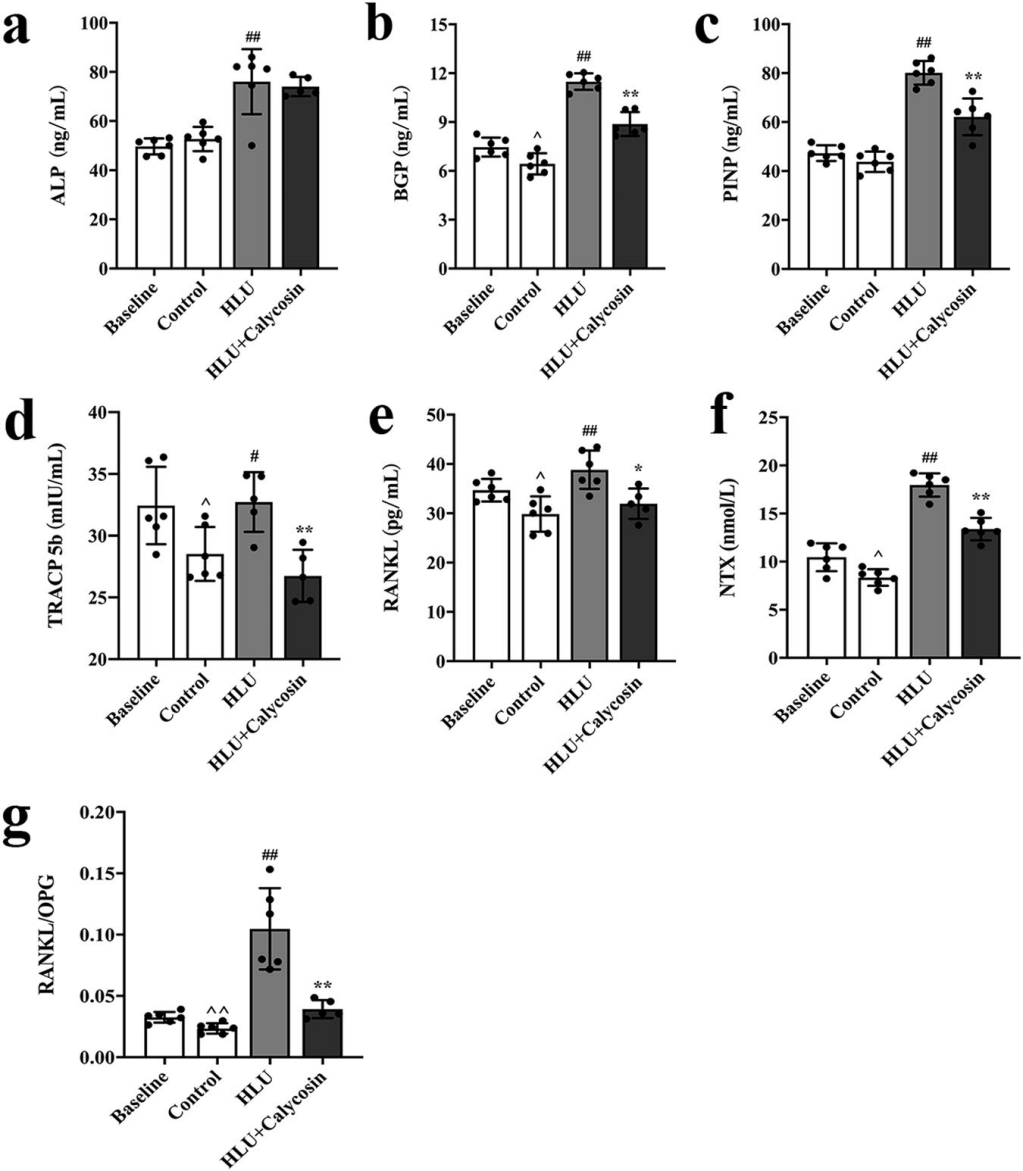
Effect of calycosin on bone turnover markers. The bone turnover biomarkers levels in rats serum of ALP (**a**), BGP (**b**), PINP (**c**), TRACP 5b (**d**), RANKL (**e**), NTX (**f**) and RANKL/OPG (**g**) in the four groups. The results are represented as the mean ± SD; *n* = 6/group. *P*-values calculated using a two-tailed Student’s *t* test (^*P* < 0.05, ^^*P* < 0.01 v.s. baseline; ^#^*P* < 0.05, ^##^*P* < 0.01 v.s. control; *P < 0.05, **P < 0.01 v.s. HLU).

The results above showed that calycosin might play an important role in maintaining bone mass of tail suspended rats. To further elucidate the underlying mechanism, the changes of the bone turnover biomarkers in the rats’ serum were detected. As shown in Fig. 5, in comparison, the serum levels of PINP, BGP, ALP, RANKL, NTX, TRACP5b and the ratio of RANKL/OPG in the HLU group significantly increased compared that in the control group (*P* < 0.01 or *P* < 0.05), indicating that the lack of load caused the bone metabolism of rats to be in a state of high bone turnover (Fig. 5a–g). However, compared with HLU rats, treatment with calycosin did not significantly alter the levels of ALP (*P* > 0.05), but obviously suppressed the HLU-induced content elevation of RANKL, PINP, BGP, NTX, TRACP5b and ratio elevation of RANKL/OPG in the serum (*P* < 0.01 or *P* < 0.05), indicating that calycosin could reduce the high bone turnover caused by tail suspension to a certain extent (Fig. 5a–g).

### Effect of calycosin on bone remodeling-related cytokines

**Fig. 6:**
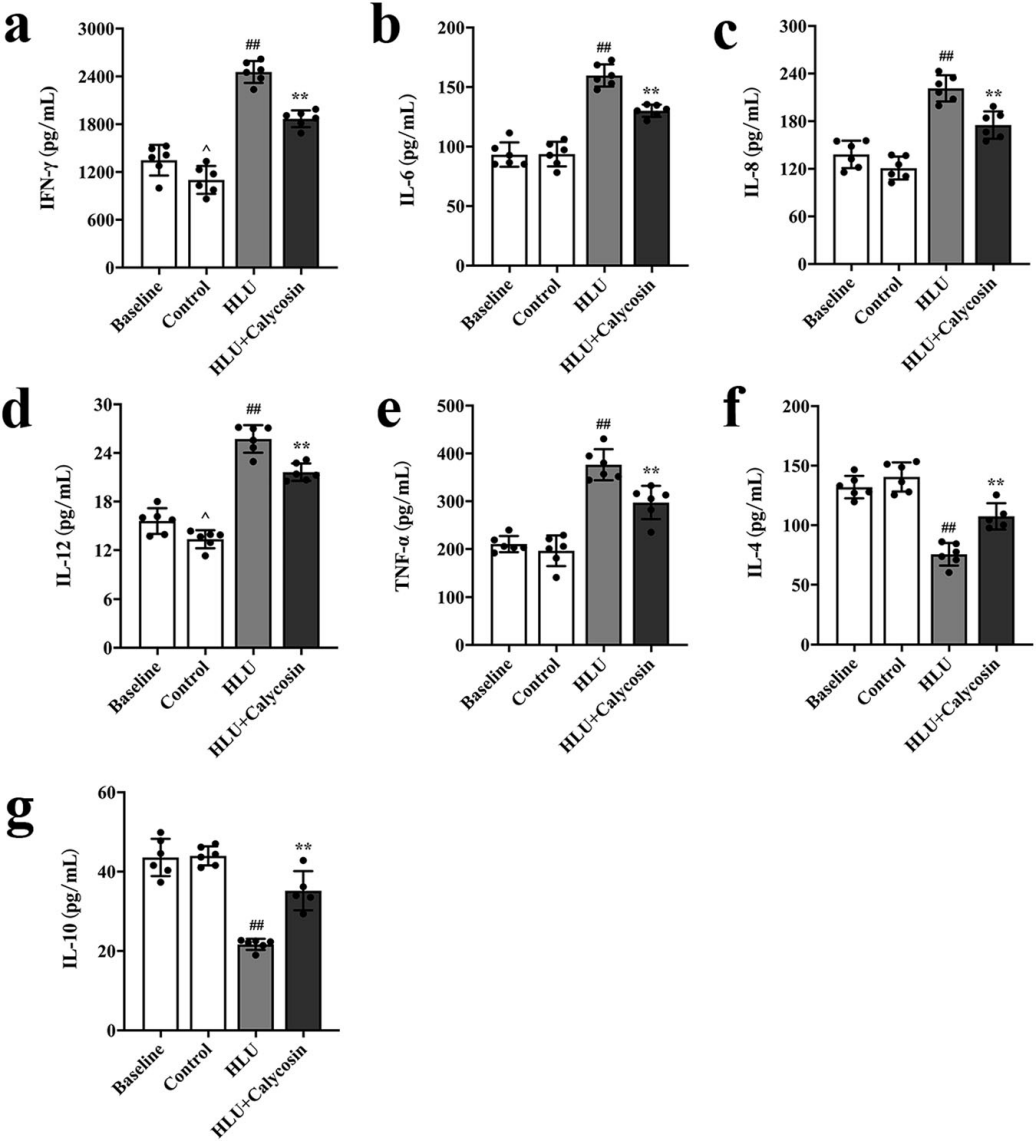
Effect of calycosin on bone remodeling-related cytokines. The levels of bone remodeling-related cytokines in rats serum IFN-γ (**a**), IL-6 (**b**), IL-8 (**c**), IL-12 (**d**), TNF-α (**e**), IL-4 (**f**) and IL-10 (**g**) in the four groups. The results are represented as the mean ± SD; *n* = 6/group. *P*-values calculated using a two-tailed Student’s *t* test (^*P* < 0.05, ^^*P* < 0.01 v.s. baseline; ^#^*P* < 0.05, ^##^*P* < 0.01 v.s. control; *P < 0.05, **P < 0.01 *v.s*. HLU).

To evaluate the effects of calycosin on cytokines related to bone remodeling, the levels of IL-4, IL-6, IL-8, IL-10, IL-12, TNF-α and IFN-γ in serum were detected using ELISA (Fig. 6). An increasing trend in the content of TNF-α, IFN-γ, IL-6, IL-8 and IL-12 was recorded in the HLU group as compared to those in the control group (*P* < 0.01), while the levels of IL-4 and IL-10 were declined (*P* < 0.01) (Fig. 6a–g). Nevertheless, treatment with calycosin significantly decreased the levels of TNF-α, IFN-γ, IL-6, IL-8 and IL-12, and increased the content of IL-4 and IL-10 notably compared with that in the HLU group (*P* < 0.01). The above results showed that calycosin administration could up-regulate the content of IL-4 and IL-10 and down-regulate the content of TNF-α, IFN-γ, IL-6, IL-8 and IL-12 in HLU rats. According to the above results, it could be seen that calycosin reduced the ratio of RANKL/OPG in serum. While IL-10, IL-12 and IFN-γ likely affect the secretion of RANKL/RANK/OPG, further regulating the process of bone remodeling. These results suggested that calycosin might regulate the process of bone metabolism through bone immunity.

## Discussion

Bone loss induced by microgravity has become one of the important threats to the health of astronauts and the major obstacle to long-term space flight. Therefore, the study aims to find potential protective drugs with low side effects for the treatment of bone loss induced by microgravity. In the present work, we preliminarily screened calycosin as a potential compound for the treatment of bone loss through the network pharmacology, and further found calycosin might play an important role in maintaining bone mass in HLU rats due to the the improvement of bone mineral density, microstructure, biomechanical properties and mineral apposition rate of bone; the effects were mainly owing to inhibit bone turnover and reduce enhanced bone remodeling by HLU. The findings may provide a potential and promising candidate in the treatment of weightless-induced bone loss.

The discovery of new small molecule drugs for weightless bone loss not only provides effective medical protection for astronauts, but also has positive significance for the development of aerospace medicine and aerospace industry. The network pharmacology provides a new idea and strategy for drug discovery. For example, Zhiguo Zhong identified quercetin as the potential ingredient of Erzhi Pill (EZP) for the treatment of bone loss through the method of system pharmacology, and further in vivo research verified that quercetin owned positive effects on osteoblast differentiation of skull^27^. This demonstrate that it is effective and feasible to discover new drugs through the method of network pharmacology. In this research, we utilized the network pharmacology approach to uncover a potential small molecule compound-calycosin for treating bone loss.

Previous studies have shown that calycosin have a dose-dependent therapeutic effect on postmenopausal osteoporosis rats and regulates the expression of OPG/RANKL *via* MAPK signaling^28^. However, there has been no direct evidence of the protective effects of calycosin on bone loss induced by microgravity in hind limb unloading rats. In this study, the Micro CT analysis showed that calycosin administration increased BMD, ameliorated the microstructure of femoral trabecular bone and increased the thickness of cortical bone in HLU rats. The MAR of bone is reflected by the distance between two injections of calcein fluorescent labeling in bone tissue. The results demonstrated that the mineral apposition rate of the bone was increased by calycosin treatment, which illustrated its role in promoting the bone formation to a certain extent. Meanwhile, the analysis of three-point bending test showed that calycosin treatment could increase the bone biomechanical parameters toughness, stiffness and Young’s modulus, but there were no significant changes in Max-load and Max-stress, indicating that calycosin administration could improve biomechanical properties to a certain degree. Taken together, these findings suggested that calycosin might play an important role in maintaining bone mass of tail suspended rats.

Aside from the well-documented development of bone loss^1^, it is also well known that there is deleterious changes in immune function and metabolic disruptions caused by spacelight^29,30^. Thence, to further investigate the underlying mechanism of calycosin in the prevention of bone loss, we detected the expressions of bone metabolism markers and bone remodeling cytokines. Serum bone turnover biomarkers can not only reflect the therapeutic efficacy of drugs that prevent bone loss, but also reveal the pathogenesis of metabolic bone. Bone turnover biomarkers include bone formation markers and bone resorption markers^31^. Among them, RANKL, NTX and TRACP5b are markers of bone resorption, and BGP, ALP and PINP are markers of bone formation. In this study, both the levels of bone resorption and bone formation markers were increased in the HLU group compared with that in the control group, which revealed that the lack of load led to an increase in bone turnover, indicating that bone homeostasis and the bone metabolism were altered in a state of high bone turnover. And the elevated levels of bone formation biomarkers in the HLU group might be due to that osteoblasts attempted to make a compensatory for bone loss induced by tail suspension. Calycosin administration obviously inhibited the increase in the serum levels of RANKL, NTX, TRACP5b, BGP, and PINP caused by HLU, whereas did not significantly change the expression of ALP. The main reason might be that there was a compensatory response in the rats body. These results above suggested that calycosin might play an important role in suppressing the high bone turnover and bone metabolism induced by HLU. Based on the results, it could be observed that calycosin administration reduced the enhanced bone remodeling caused by HLU. Recent studies have shown that the bone system can be regulated by the immune system, since both of them shared the same bone marrow microenvironment^32–35^. Specifically, the immune T cells and B cells regulate osteocytes through the secreted positive and negative cytokines, which in turn directly or indirectly regulates the coupling balance of bone formation and bone resorption, affecting bone metabolism^34–36^. For example, T helper 17 (Th-17) cells can not only induce osteoblasts to express RANKL to promote bone resorption by secreting IL-17, but also stimulate immune cells to produce IL-1, IL-6 and other inflammatory cytokines to enhance bone absorption through activating RANK signaling, resulting in bone loss^37,38^. In this study, the levels of IL-4 and IL-10 in the HLU group were decreased compared with the control group, whereas the concentration of IL-6, IL-8, IL-12, TNF-α and IFN-γ in the HLU group were increased compared with that in the control group. The changes of cytokines indicated that the lack of load might initiate the inflammatory changes of bone microenvironment state, inducing a rapid phase of bone loss. Treatment with calycosin significantly increased the level of IL-4 in serum, indicating that calycosin might induce Treg cells to produce IL-4, further regulating the balance of the bone remodeling system^39^. Calycosin significantly increased the content of IL-10, which could up-regulate the expression of OPG and down-regulate the expression of RANKL, and inhibit the differentiation and maturation of osteoclasts, further regulated bone remodeling through the pathway RANKL/RANK/OPG^40^. In addition, IL-10 also could inhibit the secretion of cytokines IL-6 and TNF-α that promoted bone resorption^41^. In general, unlike the aforementioned IL-6 and TNF-α, IL-10 is a cytokine that protects bone tissue^42^. However, the levels of bone remodeling-related cytokines in serum showed that calycosin significantly suppressed the HLU-induced elevation content of IL-6, IL-8, IL-12, TNF-α, IFN-γ that might stimulate the differentiation and maturation of osteoclasts, and promote bone resorption^43–47^. This demonstrated that calycosin might inhibit bone turnover *via* the regulation of bone remodeling cytokines. And the mechanism by which the immune system affects bone metabolism was complicated. Cytokines could directly or indirectly affect the RANKL/RANK/OPG signaling pathway, and further regulate the process of bone remodeling. The above experimental results showed that calycosin might ultimately regulated the process of bone remodeling by regulating the balance of the bone-immune system.

In summary, based on the network pharmacology, we have initially screened calycosin as a potential compound in the treatment of bone loss, and further in vivo experiments suggested that calycosin might play an important role in maintaining bone mass in HLU rats due to the improvement of bone mineral density, microstructure, biomechanical properties and mineral apposition rate of bone. And the effects were mainly related to its ability to suppress bone turnover and reduce enhanced bone remodeling caused by HLU. Therefore, calycosin might be a promising strategy to prevent bone loss and as a potential natural drug for long-term space flight.

## Supplementary information

Reporting Summary

SUPPLEMENTARY MATERIAL

## Data Availability

All data generated or analyzed during this study are included in this published article or if not are available from the corresponding author on reasonable request.
