# Supplementary material for: Calycosin prevents bone loss induced by hindlimb unloading
Source: NPJ Microgravity. 2022 Jul 6;8:23. doi: 10.1038/s41526-022-00210-x (PMC9259590; doi:10.1038/s41526-022-00210-x)
Supplement: Supplementary file 1 — Reporting Summary [file 41526_2022_210_MOESM1_ESM.pdf]

nature portfolio

Corresponding author(s): Wenjuan Zhang
Airong Qian

Last updated by author(s): Apr 2, 2022

Reporting Summary

Nature Portfolio wishes to improve the reproducibility of the work that we publish. This form provides structure for consistency and transparency in reporting. For further information on Nature Portfolio policies, see our Editorial Policies and the Editorial Policy Checklist.

Statistics

For all statistical analyses, confirm that the following items are present in the figure legend, table legend, main text, or Methods section.

n/a	Confirmed	
☐	☒	The exact sample size (*n*) for each experimental group/condition, given as a discrete number and unit of measurement
☐	☒	A statement on whether measurements were taken from distinct samples or whether the same sample was measured repeatedly
☐	☒	The statistical test(s) used AND whether they are one- or two-sided<br>*Only common tests should be described solely by name; describe more complex techniques in the Methods section.*
☒	☐	A description of all covariates tested
☒	☐	A description of any assumptions or corrections, such as tests of normality and adjustment for multiple comparisons
☐	☒	A full description of the statistical parameters including central tendency (e.g. means) or other basic estimates (e.g. regression coefficient) AND variation (e.g. standard deviation) or associated estimates of uncertainty (e.g. confidence intervals)
☐	☒	For null hypothesis testing, the test statistic (e.g. *F*, *t*, *r*) with confidence intervals, effect sizes, degrees of freedom and *P* value noted<br>*Give P values as exact values whenever suitable.*
☒	☐	For Bayesian analysis, information on the choice of priors and Markov chain Monte Carlo settings
☒	☐	For hierarchical and complex designs, identification of the appropriate level for tests and full reporting of outcomes
☒	☐	Estimates of effect sizes (e.g. Cohen's *d*, Pearson's *r*), indicating how they were calculated

Our web collection on statistics for biologists contains articles on many of the points above.

Software and code

Policy information about availability of computer code

Data collection	1. The chemical components of the medicinal herbs in Radix Astragali were identified through Traditional Chinese Medicine Systems Pharmacology Database and Analysis Platform(TCMSP).<br>2. GeneCards database was used to discover the targets of osteoporosis.
Data analysis	1. The construction and topological analysis of the network was achieved by Cytoscape 3.8.0 software.<br>2. The 3D parameters of the cancellous bone and cortical bone were analyzed by CTan software, and the 3D images of the cancellous bone and cortical bone were constructed by CTvol and CTan software in Micro-CT scanning experiment.<br>3. Matlab software was used to analyze the results in the Three-point bending mechanical test.<br>4. CaseViewer software was used to analyze the distance between two fluorescent lines in the dynamic bone histomorphometry.<br>5. GraphPad Prism 8.0 was used to statistical analyses in the manuscript.

For manuscripts utilizing custom algorithms or software that are central to the research but not yet described in published literature, software must be made available to editors and reviewers. We strongly encourage code deposition in a community repository (e.g. GitHub). See the Nature Portfolio guidelines for submitting code & software for further information.

Data

Policy information about availability of data

All manuscripts must include a data availability statement. This statement should provide the following information, where applicable:

- Accession codes, unique identifiers, or web links for publicly available datasets
- A description of any restrictions on data availability
- For clinical datasets or third party data, please ensure that the statement adheres to our policy

The data that support the findings of this study area available from the corresponding author upon reasonable request

Field-specific reporting

Please select the one below that is the best fit for your research. If you are not sure, read the appropriate sections before making your selection.

☒ Life sciences ☐ Behavioural & social sciences ☐ Ecological, evolutionary & environmental sciences

For a reference copy of the document with all sections, see nature.com/documents/nr-reporting-summary-flat.pdf

Life sciences study design

All studies must disclose on these points even when the disclosure is negative.

Sample size	The size of samples in the experiment is 6 per group. First of all, animal experiment statistics generally require at least 6 available data in each group to be meaningful, and 6 samples can meet the needs of statistical analysis. Secondly, at the beginning of the experimental design, eight samples were set up in each group. However, the rats of some groups were fell off because of tail suspension, and finally there were six samples of each group in the experiment.
Data exclusions	No data were excluded from the analyses.
Replication	The rat experiment was performed with six samples in each group, which actually ensures the repeatability of the results. And all attempts at replication were successful.
Randomization	The samples were randomly allocated into experimental groups to ensure that there was no significance difference in the body weight of the rats randomly allocated to each group statistically.
Blinding	Investigators were blinded during data collection and analysis.

Reporting for specific materials, systems and methods

We require information from authors about some types of materials, experimental systems and methods used in many studies. Here, indicate whether each material, system or method listed is relevant to your study. If you are not sure if a list item applies to your research, read the appropriate section before selecting a response.

Materials & experimental systems

n/a	Involved in the study	
☒	☐	Antibodies
☒	☐	Eukaryotic cell lines
☒	☐	Palaeontology and archaeology
☐	☒	Animals and other organisms
☒	☐	Human research participants
☒	☐	Clinical data
☒	☐	Dual use research of concern

Methods

n/a	Involved in the study	
☒	☐	ChIP-seq
☒	☐	Flow cytometry
☒	☐	MRI-based neuroimaging

Animals and other organisms

Policy information about studies involving animals; ARRIVE guidelines recommended for reporting animal research

Laboratory animals	Laboratory animals are Sprague-Dawley male rats.
Wild animals	The study did not involve wild animals.
Field-collected samples	Not applicable.

Ethics oversight	All rats were handled in accordance with the Guidelines for the Care and Use of Laboratory Animals with the approval of the Institutional Ethics Committee of Northwestern Polytechnical University.

Note that full information on the approval of the study protocol must also be provided in the manuscript.
