# Supplementary material for: Calycosin prevents bone loss induced by hindlimb unloading
Source: NPJ Microgravity. 2022 Jul 6;8:23. doi: 10.1038/s41526-022-00210-x (PMC9259590; doi:10.1038/s41526-022-00210-x)
Supplement: Supplementary file 2 — SUPPLEMENTARY MATERIAL [file 41526_2022_210_MOESM2_ESM.pdf]

Calycosin Prevents Bone Loss Induced by Hindlimb Unloading

Supplementary Information

Supplementary Tables

Supplementary Table 1. 87 compounds from the Radix Astragali

	Molecule Name	MW	OB (%)	Caco-2	DL
MOL000114	vanillic acid	168.16	35.47	0.43	0.04
MOL000131	EIC	280.5	41.9	1.16	0.14
MOL000211	Mairin	456.78	55.38	0.73	0.78
MOL001955	Heriguard	354.34	11.93	-1.03	0.33
MOL000239	Jaranol	314.31	50.83	0.61	0.29
MOL000251	Rhamnocitrin	300.28	12.9	0.48	0.27
MOL000295	alexandrin	576.95	20.63	-0.2	0.63
MOL000296	hederagenin	414.79	36.91	1.32	0.75
MOL000033	(3S,8S,9S,10R,13R,14S,17R)-10,13-dimethyl-17-[(2R,5S)-5-propan-2-yloctan-2-yl]-2,3,4,7,8,9,11,12,14,15,16,17-dodecahydro-1H-cyclopenta[a]phenanthren-3-ol	428.82	36.23	1.45	0.78
MOL000354	isorhamnetin	316.28	49.6	0.31	0.31
MOL000356	lupeol	426.8	12.12	1.46	0.78
MOL000371	3,9-di-O-methylnissolin	314.36	53.74	1.18	0.48
MOL000372	3-Hydroxy-2-picoline	109.14	62.47	1.05	0.02
MOL000373	(2S)-4-methoxy-7-methyl-2-[1-methyl-1-[(2S,3R,4S,5S,6R)-3,4,5-trihydroxy-6-methylol-tetrahydropyran-2-yl]oxy-ethyl]-2,3-dihydrofuro[3,2-g]chromen-5-one	452.5	5.38	-0.8	0.81
MOL000374	5'-hydroxyiso-muronulatol-2',5'-di-O-glucoside	642.67	41.72	-2.47	0.69
MOL000375	5'-hydroxyiso-muronulatol-2',5'-di-O-glucoside_qt	480.51	3.65	-1.11	0.8

MOL000376	7,2'-dihydroxy-3',4'-dimethoxyisoflavone-7-O-β-D-glucoside	476.47	16.16	-0.87	0.86
MOL000377	7-hydroxy-3-(2-hydroxy-3,4-dimethoxy-phenyl)chromone	314.31	5.45	0.58	0.3
MOL000378	7-O-methylisomucronulatol	316.38	74.69	1.08	0.3
MOL000379	9,10-dimethoxypterocarpan-3-O-β-D-glucoside	462.49	36.74	-0.63	0.92
MOL000380	(6aR,11aR)-9,10-dimethoxy-6a,11a-dihydro-6H-benzofurano [3,2-c]chromen-3-ol	300.33	64.26	0.93	0.42
MOL000381	13-hydroxy-9,11-octadecadienoic acid	296.5	35.6	0.44	0.17
MOL000382	Arabinose,d	150.15	1.87	-1.59	0.02
MOL000383	D-Galacturonic acid, homopolymer	194.16	29.75	-2	0.04
MOL000384	DL-Glucuronic acid	194.16	3.35	-1.91	0.04
MOL005928	isoferulic acid	194.2	50.83	0.49	0.06
MOL000386	Fucopyranose, L-	164.18	42.51	-1.32	0.03
MOL000387	Bifendate	418.38	31.1	0.15	0.67
MOL000388	gamma-aminobutyric acid	103.14	24.09	-0.26	0.01
MOL000389	FERULIC ACID (CIS)	194.2	54.97	0.53	0.06
MOL000390	daidzein	254.25	19.44	0.59	0.19
MOL000391	Ononin	430.44	11.52	-0.74	0.78
MOL000392	formononetin	268.28	69.67	0.78	0.21
MOL000393	Soyasaponin I	943.26	2.06	-2.75	0.05
MOL000394	choline	104.2	0.47	0.86	0.01
MOL000395	GGB	176.21	54.95	-0.72	0.03
MOL000396	(+)-Syringaresinol	418.48	3.29	0.47	0.72
MOL000397	cis-p-Coumarate	164.17	45.98	0.46	0.04
MOL000398	isoflavanone	316.33	109.99	0.53	0.3
MOL000399	Docosanoate	340.66	15.69	1.21	0.26
MOL000400	Flavaxin	376.41	18.18	-1.52	0.5
MOL000401	astragalosideI	869.17	46.79	-2.28	0.11
MOL000402	astragalosideI_qt	707.01	12.34	-1.15	0.2

MOL000404	astragalosideII_qt	664.97	11.55	-1.05	0.25
MOL000405	astragalosideⅢ	785.09	31.83	-2.26	0.1
MOL000406	astragalosideⅢ_qt	622.93	5.35	-1.26	0.32
MOL000407	astragalosideIV	785.09	22.5	-2.11	0.15
MOL000408	astragalosideIV_qt	622.93	7.07	-1.11	0.32
MOL000409	AstragalosideIV	785.09	17.74	-2.22	0.15
MOL000410	AstragalosideIV_qt	622.93	7.07	-0.85	0.32
MOL000411	Astraisoflavanin	464.51	18.37	-0.76	0.86
MOL000412	Mucronulatol	302.35	4.22	0.93	0.26
MOL000413	astrachrysoside A	769.09	24.55	-1.93	0.1
MOL000414	Caffeate	180.17	54.97	0.27	0.05
MOL000415	rutin	610.57	3.2	-1.93	0.68
MOL000416	Lariciresinol	360.44	5.53	0.27	0.38
MOL000417	Calycosin	284.28	47.75	0.52	0.24
MOL000418	3'-Hydroxy-4'-methoxyisoflavone-7-O-beta-D-glucoside	446.44	10.05	-0.93	0.81
MOL000419	astrasieversianin XV	901.22	11.19	-2.72	0.07
MOL000420	XLS	150.15	51.08	-1.27	0.02
MOL000421	nicotinic acid	123.12	47.65	0.34	0.02
MOL000422	kaempferol	286.25	41.88	0.26	0.24
MOL000423	rhamnocitrin-3-O-glucoside	462.44	2.87	-1.34	0.76
MOL000424	RAM	164.18	50.5	-1.21	0.04
MOL000425	asernestioside A	931.25	11.07	-3.03	0.03
MOL000426	asernestioside A_qt	769.09	24.55	-1.95	0.1
MOL000427	asernestioside B	973.29	12.54	-2.86	0.03
MOL000428	asernestioside B_qt	811.13	14.03	-1.81	0.09
MOL000429	Crystal VI	132.14	83.96	-0.88	0.02
MOL000430	betaine	117.17	40.92	-0.77	0.01

MOL000431	coumarin	146.15	29.17	1.2	0.04
MOL000432	linolenic acid	278.48	45.01	1.21	0.15
MOL000433	FA	441.45	68.96	-1.5	0.71
MOL000434	acetylastragaloside I	911.21	43.54	-2.18	0.09
MOL000435	acetylastragaloside I_qt	749.05	30.75	-1.07	0.17
MOL000436	(Z)-1-(2,4-dihydroxyphenyl)-3-(4-hydroxyphenyl)prop-2-en-1-one	256.27	87.51	0.2	0.15
MOL000437	Hirsutrin	464.41	1.86	-1.66	0.77
MOL000438	(3R)-3-(2-hydroxy-3,4-dimethoxyphenyl)chroman-7-ol	302.35	67.67	0.96	0.26
MOL000439	isomucronulatol-7,2'-di-O-glucosiole	626.67	49.28	-2.22	0.62
MOL000440	isomucronulatol-7,2'-di-O-glucosiole_qt	464.51	23.42	-0.66	0.79
MOL000441	LUPENONE	424.78	11.66	1.48	0.78
MOL000442	1,7-Dihydroxy-3,9-dimethoxy pterocarpene	314.31	39.05	0.89	0.48
MOL000054	L-	174.24	47.64	-0.49	0.03
MOL000061	Prolinum	115.15	77.57	0.22	0.01
MOL000069	palmitic acid	256.48	19.3	1.09	0.1
MOL000098	quercetin	302.25	46.43	0.05	0.28

Supplementary Table 2. Active compounds of Radix Astragali, their corresponding ADME parameters and the parameters of the compound-target network topology analysis

	Compound	OB (%)	DL	Caco-2	Structure	Degree	Neighborhood Connectivity
MOL000211	Mairin	55.38	0.78	0.73	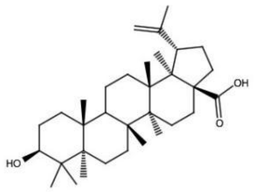	1	16
MOL000239	Jaranol	50.83	0.29	0.61	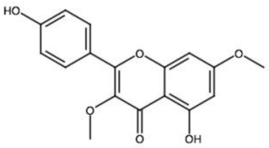	9	8.89
MOL000296	hederagenin	36.91	0.75	1.32	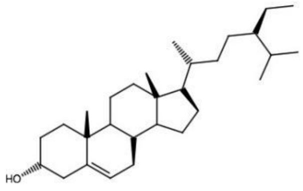	11	6.73
MOL000033	(3S,8S,9S,10R,13R,14S,17R)-10,13-dimethyl-17-[(2R,5S)-5-propan-2-yloctan-2-yl]-2,3,4,7,8,9,11,12,14,15,16,17-dodecahydro-1H-cyclopenta[a]phenanthren-3-ol	36.23	0.78	1.45	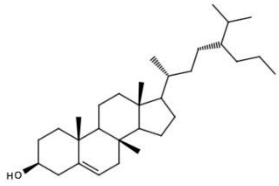	2	9.5
MOL000354	isorhamnetin	49.6	0.31	0.31	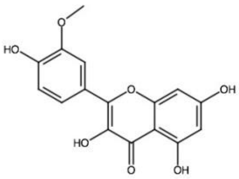	26	5.69
MOL000371	3,9-di-O-methylnissolin	53.74	0.48	1.18	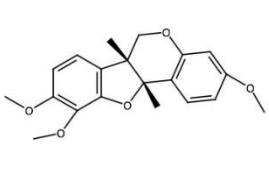	14	7.43

MOL000378	7-O-methylisomucronulatol	74.69	0.3	1.08	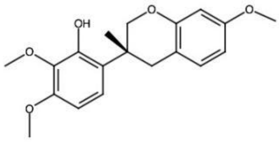	29	5.71
MOL000380	(6aR,11aR)-9,10-dimethoxy-6a,11a-dihydro-6H-benzofurano[3,2-c]chromen-3-ol	64.26	0.42	0.93	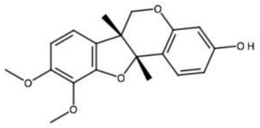	12	7.75
MOL000387	Bifendate	31.1	0.67	0.15	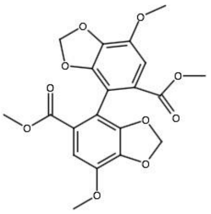	6	7.17
MOL000392	formononetin	69.67	0.21	0.78	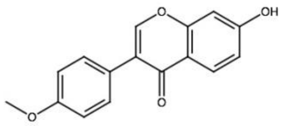	28	5.33
MOL000398	isoflavanone	109.99	0.3	0.53	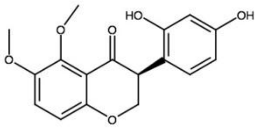	1	16
MOL000417	Calycosin	47.75	0.24	0.52	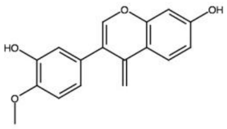	24	6.09
MOL000422	kaempferol	41.88	0.24	0.26	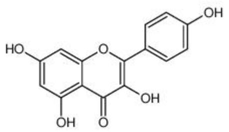	44	3.86
MOL000438	(3R)-3-(2-hydroxy-3,4-dimethoxyphenyl)chroman-7-ol	67.67	0.26	0.96	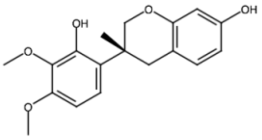	1	26
MOL000442	1,7-Dihydroxy-3,9-dimethoxy pterocarpene	39.05	0.48	0.89	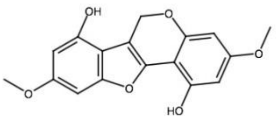	3	12

MOL000098	quercetin	46.43	0.28	0.05	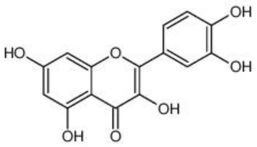	118	2.17

Supplementary Table 3. Compounds and disease targets

No.	Gene Name	Target name
1	Collagen alpha-1(I) chain	COL1A1
2	Alcohol dehydrogenase 1B	ADH1B
3	Alcohol dehydrogenase 1C	ADH1C
4	Beta-1 adrenergic receptor	ADRB1
5	Beta-2 adrenergic receptor	ADRB2
6	Aryl hydrocarbon receptor	AHR
7	RAC-alpha serine/threonine-protein kinase	AKT1
8	Arachidonate 5-lipoxygenase	ALOX5AP
9	Androgen receptor	AR
10	Apoptosis regulator BAX	BAX
11	Apoptosis regulator Bcl-2	BCL2
12	Bcl-2-like protein 1	BCL2L1
13	Baculoviral IAP repeat-containing protein 5	BIRC5
14	Caspase-3	CASP3
15	Caspase-8	CASP8
16	Caspase-9	CASP9
17	Caveolin-1	CAV1
18	C-C motif chemokine 2	CCL2
19	Cyclin-A2	CCNA2
20	G2/mitotic-specific cyclin-B1	CCNB1
21	G1/S-specific cyclin-D1	CCND1
22	CD40 ligand	CD40LG
23	Cell division protein kinase 2	CDK2
24	Cyclin-dependent kinase inhibitor 1	CDKN1A
25	Serine/threonine-protein kinase Chk2	CHEK2

26	Neuronal acetylcholine receptor protein, alpha-7 chain	CHRNA7
27	Inhibitor of nuclear factor kappa-B kinase subunit alpha	CHUK
28	Claudin-4	CLDN4
29	Collagen alpha-1(III) chain	COL3A1
30	C-reactive protein	CRP
31	Cathepsin D	CTSD
32	C-X-C motif chemokine 10	CXCL10
33	Interleukin-8	CXCL8
34	Cytochrome P450 1A1	CYP1A1
35	Cytochrome P450 1A2	CYP1A2
36	Cytochrome P450 1B1	CYP1B1
37	Cytochrome P450 3A4	CYP3A4
38	Type I iodothyronine deiodinase	DIO1
39	Dipeptidyl peptidase IV	DPP4
40	Transcription factor E2F1	E2F1
41	Pro-epidermal growth factor	EGF
42	Epidermal growth factor receptor	EGFR
43	Eukaryotic translation initiation factor 6	EIF6
44	ETS domain-containing protein Elk-1	ELK1
45	Receptor tyrosine-protein kinase erbB-2	ERBB2
46	Estrogen receptor	ESR1
47	Estrogen receptor beta	ESR2
48	Coagulation factor Xa	F10
49	Thrombin	F2
50	Tissue factor	F3
51	Coagulation factor VII	F7
52	Proto-oncogene c-Fos	FOS

53	Gamma-aminobutyric acid receptor subunit alpha-1	GABRA1
54	Gamma-aminobutyric-acid receptor alpha-5 subunit	GABRA5
55	Gap junction alpha-1 protein	GJA1
56	Glycogen synthase kinase-3 beta	GSK3B
57	Glutathione S-transferase Mu 1	GSTM1
58	Glutathione S-transferase P	GSTP1
59	Hypoxia-inducible factor 1-alpha	HIF1A
60	Heme oxygenase 1	HMOX1
61	3 beta-hydroxysteroid dehydrogenase/Delta 5-->4-isomerase type 1	HSD3B1
62	78 kDa glucose-regulated protein	HSPA5
63	Heat shock protein beta-1	HSPB1
64	5-hydroxytryptamine 2A receptor	HTR2A
65	Intercellular adhesion molecule 1	ICAM1
66	Interferon gamma	IFNG
67	Insulin-like growth factor II	IGF2
68	Insulin-like growth factor-binding protein 3	IGFBP3
69	Interleukin-10	IL10
70	Interleukin-1 alpha	IL1A
71	Interleukin-1 beta	IL1B
72	Interleukin-2	IL2
73	Interleukin-4	IL4
74	Interleukin-6	IL6
75	Insulin receptor	INSR
76	Interferon regulatory factor 1	IRF1
77	Transcription factor AP-1	JUN
78	Calcium-activated potassium channel subunit alpha 1	KCNMA1
79	Vascular endothelial growth factor receptor 2	KDR

80	Mitogen-activated protein kinase 1	MAPK1
81	Mitogen-activated protein kinase 14	MAPK14
82	Mitogen-activated protein kinase 8	MAPK8
83	Hepatocyte growth factor receptor	MET
84	Maltase-glucoamylase, intestinal	MGAM
85	Interstitial collagenase	MMP1
86	72 kDa type IV collagenase	MMP2
87	Stromelysin-1	MMP3
88	Matrix metalloproteinase-9	MMP9
89	Myeloperoxidase	MPO
90	Myc proto-oncogene protein	MYC
91	Neutrophil cytosol factor 1	NCF1
92	Nuclear receptor coactivator 1	NCOA1
93	Nuclear receptor coactivator 2	NCOA2
94	Nuclear factor erythroid 2-related factor 2	NFE2L2
95	NF-kappa-B inhibitor alpha	NFKBIA
96	Nitric oxide synthase, inducible	NOS2
97	Nitric oxide synthase, endothelial	NOS3
98	NAD(P)H dehydrogenase [quinone] 1	NQO1
99	Nuclear receptor subfamily 1 group I member 2	NR1I2
100	Nuclear receptor subfamily 1 group I member 3	NR1I3
101	Ornithine decarboxylase	ODC1
102	Delta-type opioid receptor	OPRD1
103	Mu-type opioid receptor	OPRM1
104	Poly [ADP-ribose] polymerase 1	PARP1
105	Procollagen C-endopeptidase enhancer 1	PCOLCE
106	CGMP-inhibited 3',5'-cyclic phosphodiesterase A	PDE3A

107	Progesterone receptor	PGR
108	Phosphatidylinositol-4,5-bisphosphate 3-kinase catalytic subunit, gamma isoform	PIK3CG
109	cAMP-dependent protein kinase inhibitor alpha	PKIA
110	Tissue-type plasminogen activator	PLAT
111	Urokinase-type plasminogen activator	PLAU
112	Serum paraoxonase/arylesterase 1	PON1
113	NADPH--cytochrome P450 reductase	POR
114	Peroxisome proliferator-activated receptor alpha	PPARA
115	Peroxisome proliferator-activated receptor gamma	PPARG
116	mRNA of PKA Catalytic Subunit C-alpha	PRKACA
117	Protein kinase C beta type	PRKCB
118	Phosphatidylinositol-3,4,5-trisphosphate 3-phosphatase and dual-specificity protein phosphatase PTEN	PTEN
119	Prostaglandin E2 receptor EP3 subtype	PTGER3
120	Prostaglandin G/H synthase 1	PTGS1
121	Prostaglandin G/H synthase 2	PTGS2
122	mRNA of Protein-tyrosine phosphatase, non-receptor type 1	PTPN1
123	Glycogen phosphorylase, muscle form	PYGM
124	Retinoblastoma-associated protein	RB1
125	Transcription factor p65	RELA
126	Protein CBFA2T1	RUNX1T1
127	Runt-related transcription factor 2	RUNX2
128	Retinoic acid receptor RXR-alpha	RXRA
129	Retinoic acid receptor RXR-beta	RXRB
130	E-selectin	SELE
131	Plasminogen activator inhibitor 1	SERPINE1
132	NAD-dependent deacetylase sirtuin-1	SIRT1

133	Solute carrier family 2, facilitated glucose transporter member 4	SLC2A4
134	Sodium-dependent dopamine transporter	SLC6A3
135	Sodium-dependent serotonin transporter	SLC6A4
136	Antileukoproteinase	SLPI
137	Superoxide dismutase [Cu-Zn]	SOD1
138	Osteopontin	SPP1
139	Signal transducer and activator of transcription 1-alpha/beta	STAT1
140	Estrogen sulfotransferase	SULT1E1
141	Transforming growth factor beta-1	TGFB1
142	Thrombomodulin	THBD
143	Tumor necrosis factor	TNF
144	DNA topoisomerase 1	TOP1
145	Cellular tumor antigen p53	TP53
146	Vascular cell adhesion protein 1	VCAM1
147	Vascular endothelial growth factor A	VEGFA
148	Xanthine dehydrogenase/oxidase	XDH
